# Community-Engaged Medical Student Elective to Improve Knowledge and Attitudes Toward Individuals With Intellectual and Developmental Disabilities

**DOI:** 10.7759/cureus.91126

**Published:** 2025-08-27

**Authors:** Kathryn J Kemere, Nital Appelbaum, Priya Chandan, Elisha Acosta

**Affiliations:** 1 Department of Internal Medicine, Section of Transition Medicine, Baylor College of Medicine, Houston, USA; 2 Department of Education, Innovation, and Technology, Baylor College of Medicine, Houston, USA; 3 Department of Neurological Surgery, Division of Physical Medicine and Rehabilitation, University of Louisville, Louisville, USA

**Keywords:** ableism, across the lifespan, clinical elective, community-engaged research, developmental/intellectual disability, primary care

## Abstract

Introduction: Individuals with intellectual and developmental disabilities (IDD) face multiple barriers to inclusion throughout life. One barrier is the medical knowledge and biases of adult healthcare providers with diagnoses traditionally associated with childhood. Medical school curricula have limited content related to IDD, and students have limited opportunities to engage with community organizations during their clinical years.

Methods: We created a one-month clinical elective to address medical students’ attitudes and knowledge regarding individuals with IDD using experiential learning. Students saw adult patients in our primary care clinic with a variety of IDD diagnoses and spent 30% of their time at community sites engaging with individuals with IDD in nonmedical settings. A survey of 38 attitudinal questions (Likert-scale) and a 48-item knowledge exam were administered before and after the elective.

Results: Of the 47 participants, 42 (89%) completed the paired instruments between 2018 and 2022. Of the attitudinal items, nine resulted in a more positive direction post-elective (p < 0.05). Respondents indicated receiving adequate training so that they felt increased comfort, p < 0.001, r = 0.86 (large effect), and more competent to care for a person with IDD (p < 0.001, r = 0.85). Based on a composite score of a 48-question knowledge exam, post-scores were higher than pre-scores (p < 0.001) and Cohen’s d = 0.90 (large effect).

Conclusion: The creation and implementation of a medical student elective that combines clinical experience with community engagement had a significant impact on knowledge and attitudes toward individuals with IDD. Community engagement should be considered as a mechanism to enhance other clinical electives and break down attitudinal barriers.

## Introduction

Individuals with intellectual and developmental disabilities (IDD) are a growing adult population in the United States. However, despite gains in lifespan, adults with IDD are more likely to report poor health and less likely to receive routine clinical and preventative care [1,2]. Healthcare transition and care across the lifespan for adults with IDD is being widely recognized as a priority [3]. The reality is that the medical system is coming up short in supporting this vulnerable population at a crucial time [4], and the stakes are high: increased morbidity and mortality, increased utilization of acute care services, and missed vocational opportunities are all seen during late adolescence and young adulthood [5-9]. The reasons for these system-wide shortcomings are complex and multifactorial: the individuals themselves often have medical, behavioral, and social complexity leading to a more multidimensional and complicated healthcare status; the pediatric healthcare system may not adequately prepare the individual and/or their caregivers for the numerous changes that take place upon adulthood; and adult healthcare providers often lack experience, knowledge, and confidence in providing care across the lifespan and during the vulnerable transition period. A recent study even highlighted some experienced providers’ negative attitudes toward care for those with disabilities as a major barrier to quality care across the lifespan [10]. 

These deficiencies and attitudes largely stem from the current lack of training in caring for people with disabilities, both within national medical school and residency curriculum standards. The lack of training standards leads to a paucity of physicians who are trained to address the specific care needs and to identify the barriers to optimal care for people with disability; it also increases ableism and disability bias in the healthcare system [11]. In a 2017 survey of US medical education deans, 52% of respondents reported having disability awareness as part of their medical curriculum. However, the most common format was to have people with disabilities or caregivers speak in a large group setting, and programs were mostly focused on physical disability. Nearly half of the schools without a disability curriculum expressed interest in adopting such programs but acknowledged time constraints and a lack of disability curriculum advocates as the top barriers to implementation [12]. It comes as little surprise that in a survey of four-year medical students, they reported that their education regarding people with disabilities had been inadequate, which resulted in feeling uncertain about obtaining a history, performing a physical exam, and helping patients with disabilities navigate their healthcare [13]. 

Prior research has shown that both online and in-person two-month undergraduate courses on disability care can significantly improve medical students’ knowledge and attitudes toward people with disability [14]. Even a two-hour small group session for first-year medical students introducing the ideas of ableism, disability history, and disparities increased students’ understanding of these topics and confidence in assessing barriers to care for their patients with disabilities [15]. 

Literature also shows that medical students' attitudes toward medically underserved populations decline during training. However, experiential community-based learning improves attitude outcomes [16]. One study demonstrated that students who directly interact with underserved populations during their service learning have better attitudinal outcomes than those who do not have direct interaction [17]. To impact knowledge and attitudes in clinical care, we designed a four-week medical student elective that provides clinical training to students in the care of this population and exposes our students to the daily lives of people with IDD in community settings. 

## Materials and methods

We offered a one-month clinical elective to advanced third- and fourth-year medical students, with the prerequisite of having completed either their internal medicine, pediatrics, or family medicine core clerkship. From January 2018 to October 2022, 47 medical students chose to enroll in this elective and spent approximately 70% of their rotation time (14 full days) in the Transition Medicine Clinic at Baylor College of Medicine, a patient-centered medical home for adults with IDD. This clinic provides primary care to adults 18 and older with IDD, as well as care management services and social work services. Common diagnoses in this clinic include Down syndrome, autism, cerebral palsy, spina bifida, and a variety of genetic disorders. Students saw patients with a variety of IDD diagnoses independently in the clinic and then alongside attending physicians. Preceptors were all primary care providers who care for adults with IDD regularly. Students also spent time with clinic staff working on care coordination and with social workers who provide important resources and navigate the complex systems that serve this patient population. During clinic time, students saw approximately 70 adult patients with IDD. 

Approximately 30% of the students' rotation time (six full days) was spent at external community sites, including day habilitation programs, residential living programs, the home of a patient with medical complexity, schools for individuals with IDD, and work placement programs. During these experiences, students received a brief orientation to the mission of the site and were instructed to participate in daily activities alongside individuals with IDD and get to know them, as opposed to simply observing their activities. External community sites were identified and visited by the elective directors, with frequent communication with learners and site directors to ensure the high quality of these visits.

Measures and analyses

To measure the efficacy of our elective on medical student attitudes, we utilized the National Inclusive Curriculum for Health Education-Medical (NICHE-MED) Initiative instrument [18]. NICHE-MED is an initiative of the American Academy of Developmental Medicine and Dentistry (AADMD) that supports medical educators in community-engaged curriculum interventions and program evaluation. Attitude items were rated on a five-point agreement Likert scale (1 = strongly disagree; 5 = strongly agree). The NICHE-MED collaborative of over 40 medical schools developed these items in 2016 and completed individual item validation in 2025 [18]. Each attitudinal item was analyzed independent of one another through Wilcoxon signed rank tests, followed up with effect size calculations (r) for significant differences. A Holm-Bonferroni correction was made to correct for multiple comparisons [19]. 

To measure medical student knowledge, we utilized the NICHE-MED knowledge instrument at the start and end of the educational intervention. The instrument comprises 48 boards style multiple-choice questions. Composite pre- and post-scores were calculated, and within-person changes were analyzed through a paired samples t-test and Cohen’s d effect size calculation. 

The Baylor College of Medicine Institutional Review Board reviewed and approved this study through the Expedited Procedures protocol number H-49759. The surveys were administered via REDCap, and results were de-identified before viewing and analysis. 

## Results

From January 2018 to October 2022, we had 47 medical students enroll and complete our four-week elective. The unique content and delivery of this elective drew medical students from other medical schools who completed it as an away elective. Over the course of almost five years, we engaged 12 different away sites and had six different expert faculty members provide bedside clinical teaching to the students. 

Of 47 participants, 42 (89% response rate) completed the paired knowledge instrument and the paired attitude survey. The average age of participants was 25.71 ± 2.45 years, with participant characteristics described in Table 1. 

**Table 1:** Participant characteristics IDD: intellectual and developmental disabilities Note: Participants were able to identify more than one race/ethnicity

Medical school cohort	
Second year (MS2)	6 (14%)
Third year (MS3)	9 (21%)
Fourth year (MS4)	27 (64%)
Gender	
Male	9 (21%)
Female	33 (79%)
Race/ethnicity	
Asian	15 (36%)
Black or African American	2 (5%)
Hispanic or Latina/o	5 (12%)
White	22 (52%)
Prior knowledge about IDD	
Nothing	1 (2%)
A little	37 (88%)
A lot	4 (10%)
Quality of relationships with people who have IDD	
Excellent	5 (12%)
Good	12 (29%)
Fair	16 (38%)
Poor	5 (12%)
Very poor	2 (5%)
Not applicable	2 (5%)
Last contact with someone with IDD	
Within the last week	7 (17%)
Within the last month	5 (12%)
Within the last six months	11 (26%)
Within the last year	11(26%)
More than one year ago	8 (19%)

Prior experience with IDD

Participants knew or had met an average of 19 ± 41 people with IDD (median = 5 people), mostly through individuals with whom they volunteered (n = 23), those who attended their school (n = 19), and members of their extended family (n = 18). Most participants (n = 23, 55%) did not receive specific training on serving patients with IDD. The average number of hours spent in training prior to the rotation was 11 ± 34 hours (median 3 hours). Most participants (n = 28, 67%) estimated that between 1% and 10% of their patients would have an IDD. 

Attitudes

Of the 38 attitudinal items, nine were statistically significant after the correction for multiple comparisons (Table 2). Respondents indicated greater agreement in receiving adequate training so that they felt comfortable taking care of people with IDD after the rotation (Z = -5.576, p < 0.001, r = 0.8, large effect size). They also rated greater agreement to feeling competent (Z = -5.486, p < 0.001, r = 0.85) and comfortable to care for a person with IDD (Z = -5.336, p < 0.001, r = 0.82). Interestingly, even in the pre-test, our students strongly agreed that people with IDD are discriminated against in healthcare (mean 4.62 out of 5, SD: 0.54), have greater unmet health needs (mean 4.45 out of 5, SD: 0.55), and have more difficulty in accessing healthcare (mean 4.36 out of 5, SD: 0.58). 

**Table 2:** Means, standard deviations and test statistics for pre-/post-changes in attitudes IDD: intellectual and developmental disabilities; ^a^post-mean rank > pre-mean rank; ^b^post-mean rank < pre-mean rank Note: Only differences that remained after a Holm-Bonferroni correction to the p-value have test-statistics reflected in this table. Instrument published under a Creative Commons License [18]

IDD attitudinal item	Pre-survey	Post-survey	Test-statistic (Z)	p-value	Effect size (r)
	Mean (standard deviation)				
I have received adequate training so that I feel comfortable caring for people with IDD	1.79 (0.84)	4.14 (0.61)	-5.576	<0.001	0.86a
I feel competent to care for a person with IDD	1.90 (0.98)	3.95 (0.58)	-5.486	<0.001	0.85a
I feel comfortable talking to a person with IDD	3.24 (0.96)	4.48 (0.51)	-5.336	p<0.001	0.82a
I feel uncomfortable when I interact with a person with IDD	2.62 (1.15)	1.60 (0.83)	-4.117	<0.001	0.64b
In general, it is easy for me to recognize if a person presents with an IDD	2.52 (0.86)	3.14 (0.81)	-3.629	<0.001	0.56a
The behaviors of people with IDD make me feel uncomfortable	2.31 (0.95)	1.71 (0.60)	-3.816	<0.001	0.59b
The appearance of people with IDD makes me feel uncomfortable	1.93 (0.75)	1.36 (0.53)	-3.87	<0.001	0.60b
It is more difficult to provide medical follow up for people with IDD as compared to the general population	3.31 (0.75)	2.79 (1.05)	-	<0.008	-
People with IDD should always be accompanied by a caregiver during medical appointments	2.71 (0.81)	2.24 (0.98)	-	<0.003	-
Problem behaviors (aggression, self-injury, etc.) exhibited by people with IDD may be caused by physical health problems	4.26 (0.70)	4.69 (0.47)	-3.662	<0.001	0.57a
I would prefer not to have a person with IDD as a patient	1.67 (0.79)	1.24 (0.53)	-	<0.003	-
I worry about my physical safety when I am caring for a person with IDD	2.00 (0.86)	1.60 (0.63)	-3.252	0.001	0.50a
People with IDD use tobacco as much as the general population	2.86 (0.68)	2.45 (0.86)	-	<0.019	-
People with IDD consume alcohol as much as the general population	3.05 (0.79)	2.67 (0.85)	-	<0.042	-
The majority of people with IDD engage in sexual activity	3.33 (0.69)	2.95 (0.76)	-	<0.003	-
It is more difficult to physically examine people with IDD as compared to the general population	3.45 (0.74)	3.12 (0.80)	-	<0.040	-
People with IDD have more problems with obesity as compared to the general population	3.60 (0.80)	3.83 (0.70)	-	<0.115	-
I am not comfortable shaking the hand of a person with IDD	1.60 (0.99)	1.38 (0.85)	-	<0.199	-
It is more difficult to diagnose a physical health problem for people with IDD as compared to the general population	4.10 (0.48)	3.88 (0.67)	-	<0.072	-
People with IDD experience discrimination in health care settings	4.62 (0.54)	4.45 (0.55)	-	<0.052	-
I feel like I am doing a good thing by working with a person with IDD	3.71 (0.71)	3.88 (0.77)	-	<0.181	-
People with IDD have less physical health problems as compared to the general population	1.57 (0.59)	1.43 (0.50)	-	<0.180	-
The majority of people with IDD have healthy eating habits	2.36 (0.62)	2.50 (0.71)	-	<0.216	-
I feel as if I am speaking to a child with I talk to an adult with IDD	2.45 (0.86)	2.33 (0.75)	-	<0.384	-
The majority of people with IDD engage in physical activity regularly	2.69 (0.81)	2.79 (0.81)	-	<0.489	-
People with IDD have less mental health problems as compared to the general population	1.74 (0.66)	1.64 (0.82)	-	<0.348	-
People with IDD can accurately describe their health symptoms	3.02 (0.90)	3.07 (0.75)	-	<0.725	-
It is more difficult to diagnose a mental health problem for people with IDD as compared to the general population	4.05 (0.54)	4.10 (0.69)	-	<0.834	-
Adults with IDD are not able to provide consent for health care or health services	2.02 (0.60)	1.98 (0.78)	-	<0.694	-
People with IDD experience more stress as compared to the general population	3.57 (0.70)	3.62 (0.70)	-	<0.655	-
The majority of doctors lack knowledge regarding caring for people with IDD	4.43 (0.59)	4.48 (0.63)	-	<0.674	-
People with IDD have more difficulty in accessing high-quality health care as compared to the general population	4.31 (0.56)	4.36 (0.62)	-	<0.617	-
Public health insurance programs, such as Medicaid, provide adequate insurance coverage to meet the health needs of the majority of people with IDD	2.17 (0.79)	2.14 (0.81)	-	<0.768	-
People with IDD experience greater unmet health needs as compared to the general population	4.45 (0.55)	4.48 (0.55)	-	<0.819	-
People with IDD should be treated exclusively by doctors who specialize in caring for people with IDD	2.00 (0.80)	2.00 (0.70)	-	<1.000	-
People with IDD take up more time of doctors and nurses as compared to the general population	3.36 (0.82)	3.36 (0.93)	-	<1.000	-
It is more difficult to obtain the medical history of a person with IDD as compared to the general population	3.36 (0.85)	3.36 (0.93)	-	<0.926	-
People with IDD have more difficulty in accessing health care as compared to the general population	4.36 (0.58)	4.36 (0.58)	-	<1.000	-

Knowledge instrument

Post-scores were higher than pre-scores, indicating knowledge gain post-intervention, t(41) = -5.84, p < .001, Cohen’s d = 0.90 (large effect size) [20]. 

## Discussion

Individuals with IDD are a medically underserved population facing numerous barriers to receiving adequate healthcare. Undergraduate medical education does not currently require training in the care of this population. However, recent literature demonstrates multiple ways in which medical students are being exposed to the care and life of those with IDD through a variety of educational modalities. Reported interventions have included individuals with lived experience in panels [21,22], home visits [23], virtual standardized exams [24], and interprofessional education [25,26]. Our elective is unique in that it combines community experiences with individuals with IDD, a home visit, and real-world clinical training alongside an interprofessional team. The medical students who complete our elective rotation spend more than 10 full days in the clinic seeing patients with IDD, completing, on average, more than 50 patient encounters. Through these experiences, our elective was directly shown to be effective in improving knowledge, attitudes, and comfort among students in caring for patients with IDD. Additionally, our elective encourages medical students to supplement time in the classroom with time in the community, highlighting the importance of community involvement and multimodal education to provide perspective for holistic patient care [13,17]. Partnering with people with lived experience is essential, and our students report significant benefit from getting to know individuals in the places where they live, work, and play.

It was encouraging to see that even prior to our rotation, the medical students recognized the discrimination and health disparities faced by people with IDD. Medical students are increasingly aware of the gaps in care; however, they do not feel comfortable or competent to take care of individuals with IDD after receiving their current curriculum prior to our rotation. 

A significant limitation of this educational intervention is the need for faculty experts in the care of individuals with IDD and a robust population of patients with IDD. Thankfully, there are over 20 clinics across the country that now provide primary care to adults with disabilities, the majority of which are affiliated with medical schools. The number and size of these clinics have rapidly expanded over the past several years to meet the growing need. Community sites also need to be identified for student visits. Many communities have organizations that serve the developmental and intellectual disability community, but relationships would need to be formed. In our experience, community sites are eager to include and expose healthcare learners to the important work they are doing. Improvement in governmental policies leading to the funding of curriculum development in the care of individuals with IDD is another important component in making educational efforts more robust across all institutions and disciplines.

Another limitation is that there are currently few validated instruments in medical education that directly assess the attitudes and knowledge surrounding the care of individuals with IDD; thus, there may not be a tool that accurately assesses the knowledge, skills, or attitudes being studied [18]. 

In the future, we plan to incorporate workplace-based assessments of our learners to evaluate communication skills and physical exam skills with patients with physical and intellectual disabilities, highlighting the ethical responsibility of providing high-quality care to all people. We also hope to track our learners longitudinally to see the durability of attitudinal changes and the incorporation of patients with IDD into their future medical practice.

## Conclusions

In both undergraduate and graduate medical education, there is a push to include healthcare of individuals with disabilities as part of future curricula. While many medical schools incorporate service learning, we hypothesize that it is particularly impactful to directly pair clinical learning with exposure to the daily life of medically underserved populations. We imagine that learning experiences could be created by pairing community engagement with clinical training to enhance the knowledge and attitudes of healthcare learners in a variety of other medically underserved populations. The clinical and experiential learning experience could be extrapolated to a variety of other important fields outside of IDD. 
